# Proprioceptive Indicators of Personality and Individual Differences in Behavior in Children With ADHD

**DOI:** 10.3389/fpsyg.2018.02325

**Published:** 2018-11-27

**Authors:** Liudmila Liutsko, Tania Iglesias, Josep Maria Tous Ral, Alexander Veraksa

**Affiliations:** ^1^Instituto Salud Global Barcelona, Barcelona, Spain; ^2^Faculty of Psychology, Lomonosov Moscow State University, Moscow, Russia; ^3^Department of Personality, Assessment and Psychological Treatments, University of Barcelona, Barcelona, Spain; ^4^Department of Psychology/Faculty of Humanities and Social Sciences, Universidad Del Norte, Barranquilla, Colombia; ^5^Faculty of Fine Arts, Universidad del Atlántico, Barranquilla, Colombia

**Keywords:** ADHD, proprioceptive diagnostic of temperament and character, individual differences, personality, temperament, fine motor behavior, reaction time, sustained attention, emotional control

## Abstract

Researchers have suggested that the link between personality traits and Attention-Deficit/Hyperactivity Disorder (ADHD) could be a crucial factor in understanding the disorder’s diatheses. The aim of our study was to contribute to research on personality differences (based on fine motor precision – a novel approach) in children with and without ADHD symptoms. The Children Sustained Attention Task (CSAT) and Proprioceptive Diagnostics of Temperament and Character (DP-TC) were administered to children with an ADHD diagnosis and age-matched controls. Correlational and ANOVA analyses were performed to see the association between the results of both tests and the groups’ performance. Correlational analysis suggests significant relationships between some personality dimensions (DP-TC) and correct detection in a sustained attention task (CSAT). Statistically significant differences were found between the groups on the personality dimensions (DP-TC), with the following characteristics for ADHD children: (a) temperamental tendency to pessimism; (b) high temperamental excitability; (c) high Emotionality, and (d) Behavioral Rigidity (meaning also less adaptation to changes in the environment, in temperament and character). Correct detection in the sustained attention test was significantly correlated with reaction time and the personality variables Style of Attention and Irritability. The results also showed high proprioceptive Emotionality and lack of emotional control in children with ADHD. This is an exploratory study, investigating for the first time the differences in personality (based on fine motor precision) and the relation of personality traits to scores in sustained attention for children with and without ADHD.

## Introduction

### Persons With ADHD: Studies of Personality and Individual Differences

Theory and research state that both temperament and personality systems can be useful to describe endogenous basic tendencies of thoughts, emotions, and behaviors in children (Caspi et al., 2005; De Pauw et al., 2009) and adults (Evans and Rothbart, 2007). However, there is still a lack of comparative empirical research that analyzes the connection between temperament and personality of children with Attention-Deficit/Hyperactivity Disorder (ADHD) (Mervielde and De Pauw, 2010). Martel and Nigg (2006) explained that some of the difficulties in diagnosing ADHD are due to the overlapping of the symptoms with normal childhood characteristics. The authors suggested a link between primary symptoms of inattentive, disorganized, and hyperactive/impulsive behaviors and certain aspects of personality and temperament. We found a similar perspective in the study of Martínez et al. (2010), which indicated that ADHD is a chronic disorder that begins in childhood and that the symptoms persist throughout life - i.e., considering the personality as relatively stable (without changes in emotional, cognitive, and behavioral patterns throughout life).

McKinney et al. (2011) pointed out that prior empirical research suggested a complex relationship between ADHD and temperament (Nigg et al., 2004) and the mature personality (Nigg et al., 2002; Martel et al., 2010). Nigg and Goldsmith (1998) explained that research in the field of temperament and ADHD had proceeded independently from one another, but studies integrating them could shine more light on our understanding of ADHD (Nigg et al., 2004).

Martínez et al. (2010) reported growing attention to the relationship between personality and ADHD from the perspective of different theoretical models of personality. For example, following the model of Costa and McCrae (1992), ADHD was correlated with high scores on the Neuroticism dimension, and low scores for Responsibility and Agreeableness (Ranseen et al., 1998; Nigg et al., 2002; Retz et al., 2004; Jacob et al., 2007; Miller et al., 2008; Gomez and Corr, 2014). Moreover, a tendency to experience negative emotions as well as emotional lability was reported to be the crucial component in most of the important models for the Neuroticism construct (Eysenck and Eysenck, 1985; Goldberg, 1990; Costa and McCrae, 1992; White, 1999). Parker et al. (2004), in their study of the link between adult ADHD symptomatology and the personality dimensions of the Five-Factor Model of personality (Costa and McCrae, 1992), also found that high Extraversion and Neuroticism were signi?cant predictors of ADHD symptomatology, as well as low Conscientiousness and Agreeableness, which were important for hyperactivity/impulsivity scores.

Studies based on the Millon (1969) Model reported moderately high scores on the Histrionics scale in the ADHD group and studies following the Cloninger personality model showed high scores in the Novelty Search and Harm Avoidance dimensions in adults with ADHD (Downey et al., 1996; Braaten and Rosen, 1997; Anckarsater et al., 2006; Jacob et al., 2007).

Martínez et al. (2010), who studied the traits of personality differences among clinical subtypes of ADHD in adults (inattention, hyperactivity/impulsivity, and combined), found that ADHD was not a homogeneous entity. It was dependent upon differences in personality traits among the three ADHD subtypes on the dimensions of Activity, Aggression, and Hostility of the Zuckerman-Kuhlman Personality Questionnaire (ZKPQ) and on the Histrionic, Narcissistic, Aggressive/Sadistic Passive/Aggressive, Borderline, and Paranoid scales of the Millon Clinical Multiaxial Inventory (MCMI-II).

Since ADHD diagnosis is not accurate before the age of four (Lahey et al., 1998), research related to temperament can help with investigation of its developmental features in infancy. Lahey and colleagues suggested that combining the knowledge of developmental and clinical sciences could clarify how the biological and socialization processes were involved, how they affect the problems of children with ADHD. ADHD diagnosis and temperament in children have rarely been studied, since such research has mainly been performed with adults (Nigg et al., 2004; Cho et al., 2008). Cho et al. (2008) showed that Korean children with greater ADHD symptoms had some correlations between temperament, character, and the main ADHD symptoms such as inattention, hyperactivity, and impulsivity.

González et al. (2012), in their study aiming to identify children’s temperament dimensions that are associated with ADHD in the school years, found that their temperament profile was described by low attentional, behavioral, and emotional self-regulation, as well as high emotional reactivity. The authors explain that a deficit in the executive functions involved in effortful control underlie this pattern of behaviors.

Tamm et al. (2012) underlined that intra-individual variability in reaction times on computerized tasks had become a central focus of cognitive research on ADHD for the previous decade. Greater variability in reaction time is also common in other groups, such as individuals with traumatic brain injury, high functioning autism, schizophrenia, bipolar disorder with psychotic symptoms, early stage Alzheimer’s dementia, and aging (Tamm et al., 2012). However, the precise psychological and neurophysiological meaning of the variable reaction time in ADHD is controversial; although more consistency was observed in the relationship between reaction time and inattention (a dysfunctional failure to maintain attentional control) and executive function (frontal lobe dysfunction) (Johnson et al., 2007; Tamm et al., 2012), that could also be reflected in the problems with organization, a part of arousal (Nigg, 1999).

Most researchers agree on the importance of studies of the relationships between personality, temperament, and ADHD. Our research aim is to study the relationship between proprioceptive individual differences in personality (temperament and character) in children with ADHD and age-matched control groups and to explore the relationship of these personality indicators to variables of the sustained attention test (reaction time, correct detection, errors). As Kagan (2005) has pointed out to predict behavior and to intervene successfully in it, it is more important to look at the behavior itself rather than to collect information about what the person thinks about him or herself. We hypothesized that the endogenous basic tendencies of thoughts, emotions, and behaviors described by Mervielde and De Pauw (2010) can be studied by looking at the role of proprioceptive indicators of temperament and character, to find specific individual differences in the behavior of children with and without ADHD. Since the proprioceptive individual differences and especially the focus on personality based on proprioceptive feedback were not studied systematically, this study is of an exploratory character.

## Materials and Methods

### Participants

One hundred and five children (37% girls), aged 7-14 years (9.5 ± 1.5) participated in the case-control study with the permission of their parents (with about a 50% split: 52 children with diagnosed ADHD, 13 of whom were not medicated; and 53 as a control group, matched in age and without showing the typical ADHD symptoms according to their parents’ and teachers’ evaluation).

Most of the experimental group sample data was collected thanks to the collaboration of the ADANA Fundació^1^, a foundation which is deeply involved in ADHD and invests much effort and interest in supporting research about this disorder.

The data were treated anonymously and confidentially, following the accepted institutional ethical committees of the participating centers for carrying out the study with consent of the children’s parents.

### Instruments

The participants took two types of tests: (a) the CSAT (Children Sustained Attention Task) (Servera and Llabres, 2004) and (b) the DP-TC (Proprioceptive Diagnostics of Temperament and Character) (Tous Ral et al., 2012b).

#### CSAT Method

To measure and assess sustained attention, we administered the Children Sustained Attention Task (CSAT), which is a version of the CPTs (continuous performance tests) that measures sustained attention capacity in childhood through a monitoring task. The performance of a subject is evaluated by direct scoring of successes (correct identifications/omissions), errors (false alarms), and reaction time (latent period to response in msecs). The CSAT has been shown to have good psychometric indices (with test–retest reliability ranging from 0.59 to 0.88) and is recommended for application to clinical studies (Servera and Cardo, 2006). As to its validity, the CSAT measures are more related to inattention and academic performance than to hyperactivity itself (Servera and Cardo, 2006).

#### DP-TC Test

##### Background and methodology

We used the proprioceptive (myokinetic) indicators of individual differences in personality (mainly endogenous or temperamental ones and those that had changed due to environmental adaptation, which reflect or are linked to character). This method goes back to Mira y López’s initial work, *Las correlaciones somáticas del trabajo mental* (somatic correlations of mental work) (Mira, 1923), some insights of which were later developed in his hypothesis of individual differences in fine motor control. These ideas were confirmed experimentally by Mira y López’s own observation of pilots’ performance (Liutsko, 2014), as well during his work with Luria’s polygraph (Luria, 1932). This all contributed to creating the method of *myokinetic psychodiagnosis* (MKP) (Mira, 1958). The tests that we included in the Proprioceptive Diagnostics of Temperament and Character (DP-TC) were statistically validated in Muiños’s (2008) PhD thesis work. The reliability coefficients were not very high (test–retest within 30 days), ranging from 0.40 to 0.67, but all were statistically significant (Tous Ral et al., 2012b; Tous Ral and Liutsko, 2014). DP-TC is a sensitive tool for measuring any slight changes that can occur in human behavior due to either external or internal factors. Human behavior is not stable (especially the emotions), but also can be influenced by many factors. For this reason, coffee consumption and medication intake, for example, were controlled prior to testing. The DP-TC method is a digital version of the MKP Mira y Lopez test (lineograms and parallels), which has fewer errors in output measures since it was digitalized (reducing the human error in measurements). The MKP method itself was used widely during the last century, mainly in exploratory and experimental studies, with more than 300 articles published about it (mostly in Portuguese and Spanish) (Liutsko, 2014).

The diagnostic meaning of the MKP results has also been explained by other researchers (e.g., Miroshnikov, 1973) and the method has been used in the observation of adaptation processes, such as in the flight of sportsmen to the Far East with a 7 h time difference from their departure point (Ezhov and Krivoshchekov, 2004). Ezhov and Krivoshchekov observed the changes in fine motor performance in both of the subjects’ hands (lineograms from the MKP by Mira y López) to see the coefficient of congruence/non-congruence between the two hands. The non-dominant hand was more stable in its performance for fine motor precision tasks, whereas the dominant hand appeared to be more reactive to changes created by the time zone shift and showed changes.

Tous Ral within this Mira y Lopez tradition, also attributes the observed measures in the non-dominant hand to temperamental factors, and those in the dominant hand to character-related factors (Tous Ral et al., 2012b; Tous Ral and Liutsko, 2014). Changes in the precision of fine motor performance can occur due to changes in the environment and/or individual differences (Tous Ral and Liutsko, 2014), stress and/or illness in patients with cancer (Liutsko et al., 2016), Parkinson’s disease (Gironell et al., 2012), multiple sclerosis (Liutsko and Tous Ral, 2013), and developmental/maturation and aging processes (Liutsko, 2014; Liutsko et al., 2014a,b). Moreover, stress created by the simultaneous performance of tasks, such as the addition of a cognitive task like counting backwards from 100, to a fine motor performance task, also affected the fine motor precision of students on the Irritability dimension of DP-TC, causing an increase in Line Length performance^2^ similar to what is observed due to aging processes (Liutsko et al., 2014c).

Tous Ral et al. (2012b) explained that proprioceptive information does not depend on the variability of our exteroceptive organs, but rather on the variability of our bodily changes, and therefore the variability of the miokinetic response of each person. The authors pointed out that the instrument may be used today in a direct assessment of temperament and character as a non-verbal tool (a test for personality assessment). The new point of this system is to assess indirectly (through movements of the hand in graphical response to stimuli) behavior also can be affected by posture and emotions. Systematic biases, measured in fine motor precision tasks, are observable in the performance of every person (Tous Ral et al., 2012a; Liutsko, 2013).

##### DP-TC method in this study

DP-TC is a graphical test based on fine motor precision (Tous Ral et al., 2012b). The test consists of tracing lines of models that appear on a touch screen, with or without visual guidance. The method was developed on the basis of the manual tests - lineograms and parallels - of Mira y López’s MKP, in different stages of digitization (Tous Ral et al., 2007, 2012b; Muiños, 2008) and validation (Tous Ral et al., 2005b). It has been used for personality and impulsivity assessment together with verbal tests, since the results complement the information obtained by other methods (Tous Ral et al., 2004, 2005a). The computerized measures of the DP-TC facilitate both direct measurements and posterior interpretation and comparison of data (raw or standardized).

*The instruction* given to participants was to trace the model lines and continue the same pattern as accurately as possible while performing all the tasks, starting with the visual-proprioceptive and followed by the proprioceptive sensory condition.

Observable variables were obtained by the DP-TC test along the following dimensions:

(1) *Irritability* – line length (LL): Change in line length (compared to the 40-mm-long model), with the corresponding indexes: LLd and LLnd, for dominant hands (character) and non-dominant hands (temperament), respectively;
(2) *Behavioral Variability/rigidity* - Line Length Variability (LLV): The variability of line length in parallels, with the corresponding indexes: LVd and LVnd, for dominant and non-dominant hands, respectively;
(3) *Mood* – directional bias (parallel to the model line) in frontal movement type, with the corresponding indexes: DFd and DFnd for dominant and non-dominant hands, respectively;
(4) *Decision Making* – directional bias in sagittal movement type, with the corresponding indexes: DSd and DSnd;
(5) *Style of Attention* – directional bias in transversal movement type, with the corresponding indexes: DTd and DTnd;
(6) *Emotionality* – formal bias (perpendicular to the model line), with the corresponding indexes: FFd and FFnd.

### Data Analysis

The descriptive statistics, ANOVA, and correlation analyses were calculated to represent the results of this study and were performed with the use of SPSS v.19.

## Results

The results of the CSAT (Children Sustained Attention Task) performance between the ADHD and control group revealed statistically significant differences in mean values for success (correct detection) (*p* = 0.019), with a medium effect size (Cohen’s *d* = -0.47), and with worse indicators for the children with ADHD, but not in errors (false identification) or reaction time (Table 1).

**Table 1:** Descriptive statistics and ANOVA differences in CSAT (Children Sustained Attention Task) between ADHD and control groups.

CSAT variables	Groups	*M*	*SD*	Cohen’s *d*	*F*(1, 103)	*p*-value
Correct detection (success)	ADHD	68.60	15.36	-0.47	**5.71**	**0.019**
	Control	75.04	12.10			
Errors (false replies)	ADHD	60.63	139.88	0.30	2.27	0.135
	Control	28.91	62.12			
Reaction time (msec)	ADHD	391.10	59.31	-0.01	0.00	0.964
	Control	391.37	6.11			

*Numbers in bold are for p-values of a significant level.*

As for the results in the fine motor precision test linked to individual Behavioral Variability (DP-TC), based on representing lines (in lineograms and parallels) in the proprioceptive condition (without visual guidance), we observed the following most significant differences between the two groups:

(1) In the directional bias, frontal movement in the non-dominant hand (temperament): a tendency toward the pessimism pole (Mood dimension) in the ADHD group;
(2) In the formal bias, frontal movement for both hands (temperament and character^3^): a tendency toward being “warmer” in affective and dependent behavior with a need to be “affiliated” in the ADHD group, or “colder” or more distant in relations with others and more independent in the control group;
(3) In Line Length Variability (parallels), both hands (temperament and character): a tendency toward more rigid behavior in the children with ADHD (Table 2).

Moreover, a difference in LL (Line Length) performance was observed in the non-dominant hand (48.90 ± 15.52 mm average value in the ADHD group vs. 43.36 ± 13.33 mm in the control group), however, this difference did not reach statistical significance (*p* = 0.052) (Table 2).

**Table 2:** Descriptive statistics and ANOVA differences in DP-TC (proprioceptive fine motor control) between ADHD and control groups.

DP-TC variables	Movement type	Groups	*M*	*SD*	Cohen’s *d*	*F*	*p*-value
Mood (pessimism – optimism)	DFnd	ADHD	-15.48	23.66	-0.41	**4.30**	**0.041**
		Control	-6.87	18.63			
	DFd	ADHD	-5.77	16.85	0.19	0.94	0.335
		Control	-9.11	18.44			
Style of Attention (intra – extra)	DTnd	ADHD	3.23	33.18	-0.06	0.08	0.777
		Control	4.75	20.30			
	DTd	ADHD	-2.85	30.68	0.03	0.02	0.878
		Control	-3.60	18.36			
Decision making (submission – dominance)	DSnd	ADHD	12.63	22.08	-0.16	0.69	0.407
		Control	15.83	17.00			
	DSnd	ADHD	13.69	20.78	-0.08	0.15	0.704
		Control	15.02	14.34			
Emotionality (distant - affectionate)	FFnd	ADHD	23.06	26.70	0.60	**9.19**	**0.003**
		Control	10.96	11.31			
	FFd	ADHD	22.77	29.40	0.67	**11.42**	**0.001**
		Control	8.60	8.13			
Irritability (inhibition – excitability)	LLnd	ADHD	48.90	15.52	0.39	3.86	0.052
		Control	43.36	13.33			
	LLd	ADHD	42.12	15.30	-0.02	0.01	0.933
		Control	42.34	11.73			
Behavioral flexibility (rigidity – variability)	LLVnd	ADHD	15.25	15.31	-0.65	**10.77**	**0.001**
		Control	22.85	7.00			
	LLVd	ADHD	18.96	15.56	-0.56	**8.11**	**0.005**
		Control	25.62	6.85			

*Indexes nd – non-dominant hand (corresponds to temperament variables in the DP-TC) and d – dominant hand (corresponds to character variables in the DP-TC). Numbers in bold are for p-values of a significant level. Alpha = 0.05.*

The correlation analysis when comparing the two groups (the column “all” in Table 3), between the observable variables of CSAT/CSAT and CSAT/DP-TC tests, showed that:

**Table 3:** Correlational analysis between CSAT and DP-TC tests.

CSAT variables		Correct detection			Errors			Reaction time		
Groups		ADHD	Control	All	ADHD	Control	All	ADHD	Control	All
**CSAT**								
Correct detection		1	1	1						
Errors		-0.10	-0.27	-00.17	1	1	1			
Reaction time		**-0.56^∗∗^**	0.06	**-0.26^∗∗^**	-0.07	-0.14	-0.09	1	1	1
**DP-TC**								
Mood	DFnd	-0.10	0.12	-0.06	-0.10	0.13	-0.06	0.25	0.00	0.13
(Pessimism – optimism)	DFd	-0.05	0.04	-0.03	-0.06	-0.24	-0.09	0.08	0.09	0.09
Style of Attention	DTnd	-0.19	-0.01	-0.12	-0.10	-0.19	-0.12	0.09	0.08	0.08
(Intra – extra)	DTd	0.14	**0.28^∗^**	**0.18^∗^**	-0.06	-0.22	-0.09	-0.08	0.06	-0.03
Decision making	DSnd	-0.26	-0.06	-0.16	0.07	-0.18	-0.01	-0.05	-0.10	-0.07
(Submission – dominance)	DSd	-0.14	-0.18	-0.14	0.04	0.08	0.04	0.02	-0.02	0.01
Emotionality	FFnd	0.01	0.01	-0.06	-0.09	-0.10	-0.05	-0.07	0.02	-0.04
(Distant – affectionate)	FFd	0.01	0.05	-0.06	-0.03	0.01	0.02	-0.01	-0.27	0.04
Irritability	LLnd	-0.23	-0.25	**-0.27^∗∗^**	0.10	0.14	0.13	0.09	-0.20	-0.05
(Inhibition – excitability)	LLd	**-0.34^∗^**	-0.17	**-0.26^∗∗^**	-0.08	-0.04	-0.07	0.13	0.06	0.10
Impulsivity/Variability (rigidity – variability)	LLVnd	0.01	-0.11	0.05	0.16	-0.07	0.07	0.05	-0.20	-0.03
	LLVd	0.06	0.05	0.11	0.15	-0.01	0.08	0.11	0.11	0.10

*p-values with statistical significance at ^∗^ < 0.05 and ^∗∗^ < 0.01.*

(1) Within the CSAT test variables, a negative relationship exists between the frequency of committing errors (false identifications) and correct identifications, as well as between reaction time and correct identification (indicator of good sustained attention);
(2) Style of Attention (in using the dominant hand), a dimension of the DP-TC test, had a weak but statistically significant relationship with correct detection (good sustained attention) of the CSAT test; and
(3) The Irritability dimension of the DP-TC was related to the correct identification (good sustained attention) of the CSAT test for both hands, statistically significant, with a negative sign (Table 3).

In order for the correlations to be distributed normally, we also performed the Fisher r-to-z transformation of Pearson’s r, according to the formula:

z_r_ = (1/2)[log_e_(1+r) – log_e_(1–r)] and standard error: SEz_r_ = 1/sqrt[N-3] online calculator, available at: http://vassarstats.net/tabs_rz.html)

The results are given in Table 4.

**Table 4:** Fisher’s r-to-z between CSAT and DP-TC tests.

CSAT variables		Correct detection			Errors			Reaction time		
Groups		ADHD	Control	All	ADHD	Control	All	ADHD	Control	All
**CSAT**				
Correct detection		1	1	1						
Errors		-0.10	**-0.28**	-00.17	1	1	1			
Reaction time		**-0.63**	0.06	**-0.27**	-0.07	-0.14	-0.09	1	1	1
**DP-TC**				
Mood	DFnd	-0.10	0.12	-0.06	-0.10	0.13	-0.06	0.25	0.00	0.13
(Pessimism – optimism)	DFd	-0.05	0.04	-0.03	-0.06	-0.24	-0.09	0.08	0.09	0.09
Style of Attention	DTnd	-0.19	-0.01	-0.12	-0.10	-0.19	-0.12	0.09	0.08	0.08
(Intra – extra)	DTd	0.14	**0.29**	**0.18**	-0.06	-0.22	-0.09	-0.08	0.06	-0.03
Decision Making	DSnd	-0.26	-0.06	-0.16	0.07	-0.18	-0.01	-0.05	-0.10	-0.07
(Submission – dominance)	DSd	-0.14	-0.18	-0.14	0.04	0.08	0.04	0.02	-0.02	0.01
Emotionality	FFnd	0.01	0.01	-0.06	-0.09	-0.10	-0.05	-0.07	0.02	-0.04
(Distant – affectionate)	FFd	0.01	0.05	-0.06	-0.03	0.01	0.02	-0.01	-0.27	0.04
Irritability	LLnd	-0.23	-0.26	**-0.28**	0.10	0.14	0.13	0.09	-0.20	-0.05
(Inhibition – excitability)	LLd	**-0.35**	-0.17	**-0.27**	-0.08	-0.04	-0.07	0.13	0.06	0.10
Impulsivity/Variability (rigidity – variability)	LLVnd	0.01	-0.11	0.05	0.16	-0.07	0.07	0.05	-0.20	-0.03
	LLVd	0.06	0.05	0.11	0.15	-0.01	0.08	0.11	0.11	0.10

*Numbers in bold are for p-values of a significant level.*

The corresponding standard errors (SEz_r_) are the following: per each group (ADHD and control), both are rounded to 0.14, and in general (both together), 0.099.

If we consider the analysis by each group separately, we observe that the children from the ADHD group have stronger relationships with a negative sign between correct detection (CSAT) and reaction time (CSAT), Irritability dimension (excitability, DP-TC), and Decision-Making (dominance or aggressiveness, DP-TC); whereas the children from the control group showed a significant relationship with a positive sign for Style of Attention (Extratension, DP-TC) (Tables 3, 4).

## Discussion

### Descriptive and ANOVA Analysis

While the relationships between temperament and character or personality and the severity of ADHD symptoms have been studied mainly in adult populations (Nigg et al., 2004), our study contributes to research on the situation with children. This study is novel due to its methodology (based on fine motor precision performances) and provides a new outlook for the understanding of ADHD from the standpoint of individual differences in personality (temperament and character). Children with ADHD differ among themselves as well as being similar to children without ADHD, which could create controversies in ADHD diagnosis, as mentioned by Martel and Nigg (2006), either because there would be little homogeneity (Martínez et al., 2010), since we observed high within-group variability in responses reflected in *SD*s, which may be considered controversial and a study limitation. However, this provides information that the children with ADHD are much more variable in their behavior than the control group, whose members are more similar in their fine motor behavior and corresponding personality dimensions (the basis of the DP-TC).

Higher variability within the ADHD group was also observed in the performance of the CSAT test. If we compare just mean values for errors (false identifications) in the CSAT, there is a difference between the average values for each group’s performance: 61 (ADHD) vs. 29 (control). However, the variance was greater within the ADHD group, showing that this group included participants whose CSAT points ranged from very low (compared with controls) to very high, meaning that some responders from the ADHD group could have results comparable to or even better than the control group. This concerned only a very limited number of children, since the average results for the ADHD group were worse than the average results of the control group.

A similar situation for intra-group variability (in ADHD only) was observed for reaction time (CSAT), which is congruent with many other studies’ results for reaction time and attention (e.g., Johnson et al., 2007) and other cognitive tasks (reviewed by Tamm et al., 2012). In our study, the mean values for the two groups are almost identical; the variance for the ADHD group is higher. This point makes it more difficult to compare them quantitatively; nevertheless, it is important for qualitative observation. For this reason, it makes sense to describe the ADHD group as diverse in their individual behaviors for some observable variables: in the CSAT test for reaction time and errors (false identifications), whereas correct detection (good sustained attention) was quite homogeneous in terms of within-group individual variability.

Our results obtained from the DP-TC test confirm the idea of other authors about personality differences in children with and without ADHD (Parker et al., 2004). In terms of adaptive vs. less adaptive behavior in children with ADHD, we can see the changes from more stable (temperamental) features to more flexible ones (character), which show adaptation processes, as the DP-TC variables changed in performance by the non-dominant hand (corresponding to more stable or temperamental characteristics) vs. the dominant hand (corresponding to character or reflecting behavior more reactive to environmental changes) (Ezhov and Krivoshchekov, 2004; Tous Ral et al., 2012b).

The tendency for pessimism on the Mood dimension of the DP-TC was shown in the children from the ADHD group only with the non-dominant hand (-15.48 ± 23.66 mm) if comparing both the dominant one and average values of the control group in both hands). This suggests a more temperamental, endogenous, or biological tendency to pessimism, confirming the idea of Caspi et al. (2005) about endogenous temperamental tendencies in the emotions and behavior of children with ADHD. However, this is balanced in the ADHD group, since there were no significant differences observed for the dominant hand (character or more “state” features). These results suggest that for these children (or at least those in the ADHD group), their endogenous temperamental tendency to pessimism was controlled in their current behavioral state.

A similar tendency was also observed for Irritability (inhibition-excitability), which was higher in the non-dominant hand (temperament). In raw average values: Line Length performance was 48.90 ± 15.52 mm in the ADHD group in the non-dominant hand vs. 42.12 ± 15.30 mm in the dominant hand, which was closer to the control group’s performance (42.34 ± 11.73 mm in the dominant hand vs. 43.3 ± 13.33 mm in the non-dominant hand), and model Line Length itself (40 mm). Here the tendency to higher endogenous excitability in ADHD children was also somehow adapted, as per the results of the “reactive” hand performance. In both cases we can speak of adaptive, favorable processes that occurred due to education, therapy, and/or medication. Moreover, high excitability does not always have a negative interpretation: People with a high IQ were also found with it (Liutsko, 2014).

Nevertheless, two other differences, most significant since they were big and statistically significant, observed between the groups did not have such adaptive changes, since similar results persisted in both hands on the DP-TC dimensions of Emotionality and Behavioral Variability. Greater emotional lability was shown in the ADHD group, indicating congruence with the observations and results mentioned by other authors who mentioned emotional lability and low maturity (compared with a control group of similar ages) in persons with ADHD (Nigg et al., 2002; Martel et al., 2010; González et al., 2012). On the dimension of Behavioral Variability, the results revealed more rigid patterns in the ADHD group average for each group values on the dimension, though with greater variability in individual performance within the same group, indicating non-homogenous individual performance and behavior of these children. Both of these dimensions (Emotionality and Behavioral Rigidness/Flexibility) are important to consider in treatment to reduce the symptoms of ADHD, since they were not shown to have been modified yet by educational or medical interventions (significant differences were shown for both hands).

### Correlational Analysis

With regard to correlation analysis, although reaction time (latent period to reply) was almost identical in the two groups, it had a significant relationship (*p* < 0.001) to the number of correct answers on the CSAT test in the ADHD group only. However, reaction time had no significant relationship in the control group for correct answers, and thus was not so important. This suggests that only the ADHD children, when they respond more quickly, give more correct responses due to a negative sign relationship, and vice versa – the longer they take to respond, the less correct are their responses. Possibly in this context, the longer time spent by the children from the ADHD group on answering (longer latent period to reply) could be related to greater distraction or loss of motivation, with resulting worse scores on the CSAT test. Alternatively, sustained attention and better concentration might be maintained for a short time, while getting worse as the time increased. No similar studies have been performed in this context (showing the relationship between latent response time and correct answers in the children with ADHD), so our results could be considered as the first step in this direction.

A weak but statistically significant relationship was observed only in the control group between Style of Attention (DP-TC) and correct responses (CSAT), meaning that the more attention was paid to the external world, the larger the number of correct responses on the sustained attention test. This tendency was not as pronounced in the children with ADHD.

Another important tendency that can be considered as an indicator of more pronounced temperamental submission (or less dominance and aggressiveness, DP-TC) in the ADHD group might be related to an increase in correct detection (an indicator of good sustained attention) in the CSAT test (although this correlation did not reach a statistically significant value, *p* = 0.052, *r* = -0.26).

Since the Irritability dimension (DP-TC) had a weak, but statistically significant relationship (with a negative sign in both groups) with correct detection on the CSAT, it would be a considerable general feature, however, with more weight (due to a little bit higher correlation value) in the adaptive behavior (dominant hand) of the children with ADHD. Thus, less excitability/more inhibition would be reflected in a higher percentage of correct answers on the CSAT.

No statistically significant relationship was observed between the dimension of Variability in Behavior (DP-TC) and the indicators of good sustained attention, showing that this feature is just an indicator of individual differences between the ADHD and control groups, but this does not greatly influence the results of the sustained attention test in this study.

### Limitations

We analyzed data in the full subgroups, otherwise the sample size would have been reduced significantly, and for this first exploratory study, the aim was to observe the general situation in children. But this approach has several limitations that should be considered when interpreting or generalizing the results. The first is that the range age is quite big and children in the preadolescent period can differ from those who are younger. The second limitation is that we did not have an opportunity to split the groups into other, smaller subgroups, such as different subtypes of ADHD or those children taking medication, although we controlled for the time of medication uptake and the tests were performed when the least effects of medication were seen (before the next uptake). Finally, the control group consisted of children selected by teachers as “without visible ADHD symptoms or tendencies”; they generally also had better academic performance and motor coordination than the children with ADHD. Some of the participants in the control group were attending musical or dance classes, which could have been a reason for better proprioceptive motor precision compared to those with ADHD. Thus, further studies are needed with a more homogenous control group, or split by design into those who had better and worse academic and motor performance. Also it would be appropriate to see whether the music or dance classes could be beneficial as an alternative therapy for children with ADHD.

## Conclusion

Significant individual differences in behavior were observed between the ADHD children and the control group, based on the Proprioceptive Diagnosis of Temperament and Character (DP-TC). Two variables – Mood (endogenous or temperamental tendency to pessimism) and Irritability (endogenous or temperamental tendency to excitability) in the performance of the group with ADHD were shown to be more adaptive, since these tendencies were not pronounced in the subject’s character (dominant hand) and were similar to the control group’s average results. On the other hand, two DP-TC dimensions, Emotionality (higher Emotionality in the ADHD group) and Variability in Behavior (higher rigidness in the average value, but with wider dispersion in interval of individual performance in the ADHD group) were non-adaptive, since the performance persisted at the same level in both hands (non-dominant and dominant) and with a statistically significant difference from the control group. The correlational analysis of the individual differences revealed that not all differences were important for the quality of performance of the sustained attention task, supporting mainly a significant relationship with Irritability (balanced excitability/inhibition) among all of them.

This is an exploratory study and requires more research along these lines and exploration of the Emotionality dimension in children with ADHD. High Emotionality can induce more physical movement among children, which could compensate or be a part of therapy instead. This study is a pioneer in the context of proprioceptive indicators (individual differences in personality) of children with ADHD and the control group of children who have not been so diagnosed, and have better academic as well as motor performance. The results could provide an orientation in work with children who are diagnosed with ADHD. Moreover, physical activity and music or art therapy for children with ADHD could be considered means of “emotional discharge,” to help them cope with their emotions, however, more studies with this focus are needed.

## Ethics Statement

This study was carried out in accordance with the recommendations of Ethical committee of the University of Barcelona with written informed consent from all subjects (parents of the childrens involved in the study) in accordance with the Declaration of Helsinki. The protocol was approved by the Ethical committee of the University of Barcelona and the educational centers (the Europa International School and the Rosa dels Vents school in Barcleona, Spain).

## Author Contributions

LL and JR made substantial contributions to the conception or design of the work or the acquisition. JR proved the study at the Ethics Committee of the University of Barcelona. TI recruited volunteers, performed all the testings, contributed to the literature review and writing the Introduction part, and reviewed and proved the manuscripts’ drafts. LL analyzed and interpreted the data for the study, and drafted the work. AV revised the draft critically for important intellectual content, approved the final version to be published,œ and agreed to be accountable for all aspects of the work in ensuring that questions related to the accuracy or integrity of any part of the work are appropriately investigated and resolved.

## Conflict of Interest Statement

The authors declare that the research was conducted in the absence of any commercial or financial relationships that could be construed as a potential conflict of interest.

## Footnotes

1 The ADANA Foundation in Barcelona, founded in 1997, is non-profit foundation whose aim is to improve the quality of life of persons with ADHD. It helped in this study by recruiting volunteers with ADHD. Other schools that helped to provide a control group are listed in the Acknowledgments.

2 Tracing (with vision) and drawi ng lines by proprioceptive or myokinetic memory (without vision).

3 For the children, since their character is still in formation, when we refer to their “character,” we mean the changes in their personality due to education/environmental effects and their development.
